# Secondary Budd-Chiari syndrome occurred after adjuvant radiotherapy for perihilar cholangiocarcinoma: a case report

**DOI:** 10.1186/s12957-023-02890-5

**Published:** 2023-01-16

**Authors:** Yuya Miura, Ryo Ashida, Atsushi Saiga, Teiichi Sugiura, Katsuhisa Ohgi, Mihoko Yamada, Shimpei Otsuka, Takeshi Aramaki, Rui Sato, Katsuhiko Uesaka

**Affiliations:** 1 Division of Hepato-Biliary-Pancreatic Surgery, Shizuoka Cancer Center, 1007 Shimonagakubo, Nagaizumi-cho, Sunto-gun, Shizuoka, Japan; 2 Division of Interventional Radiology, Shizuoka Cancer Center, 1007 Shimonagakubo, Nagaizumi-cho, Sunto-gun, Shizuoka, Japan

**Keywords:** Budd–Chiari syndrome, Adjuvant radiotherapy, Stenosis of the hepatic vein, Radiation-induced stenosis of the hepatic vein, The side effect of radiation, Stenting for the hepatic vein

## Abstract

**Background:**

Budd–Chiari syndrome (BCS) is a rare vascular disorder of the liver, and acute and secondary BCS is even rarer.

**Case presentation:**

A 62-year-old man with perihilar cholangiocarcinoma of Bismuth type IIIa underwent right hemi-hepatectomy with caudate lobectomy and pancreatoduodenectomy. Adjuvant chemoradiotherapy was performed due to a positive hepatic ductal margin. Subsequently, the disease passed without recurrence. The patient visited for acute onset abdominal pain at the 32nd postoperative month. Multidetector-row computed tomography (MDCT) showed stenosis of the left hepatic vein (LHV) root, which was the irradiated field, and thrombotic occlusion of the LHV. The patient was diagnosed with acute BCS caused by adjuvant radiotherapy. Although anticoagulation therapy was performed, the patient complained of sudden upper abdominal pain again. MDCT showed an enlarged LHV thrombus and hepatomegaly. The patient was diagnosed with exacerbated acute BCS, and stenting for the stenotic LHV root was performed with a bare stent. Although stenting for the LHV root was very effective, restenosis occurred twice due to thrombus in the existing stent, so re-stenting was performed twice. The subsequent clinical course was acceptable without recurrence or restenosis of the LHV root as of 6 months after the last stenting using a stent graft.

**Conclusion:**

Although no case of BCS caused by radiotherapy has yet been reported, the present case showed that late side effect of radiotherapy can cause hepatic vein stenosis and secondary BCS.

## Background

Curative resection is an indispensable treatment for biliary tract cancer [1], but a positive ductal margin has been identified as one of the most important risk factors for the recurrence of cholangiocarcinoma after radical surgery [2, 3]. Several efforts to improve the survival have been conducted through perioperative multimodal therapy, including adjuvant radiotherapy with surgical resection, especially in cases with positive resection margins [1, 2]. However, complications associated with radiotherapy for perihilar cholangiocarcinoma are not well discussed due to the small number of cases.

Budd–Chiari syndrome (BCS) is a rare vascular disorder of the liver caused by obstruction anywhere from the root of the hepatic vein to the inferior vena cava (IVC) [4], and it has several clinical presentations depending on the mechanism underlying the impaired venous outflow [5]. Acute BCS is rarer than chronic BCS and presents with painful hepatomegaly, ascites, and hepatic insufficiency [5]. In addition, secondary BCS, which is caused by invasion or extrinsic compression of the hepatic vein and/or the IVC, is rarer than primary BCS, which is associated with hypercoagulable states leading to vascular thrombosis [5]. Although secondary BCS is usually caused by vascular compression due to liver tumor [5], a case caused by vascular stenosis due to radiotherapy has not yet been reported.

We herein report a case of secondary and acute BCS caused by adjuvant radiotherapy for perihilar cholangiocarcinoma.

### Case presentation

**Fig. 1:**
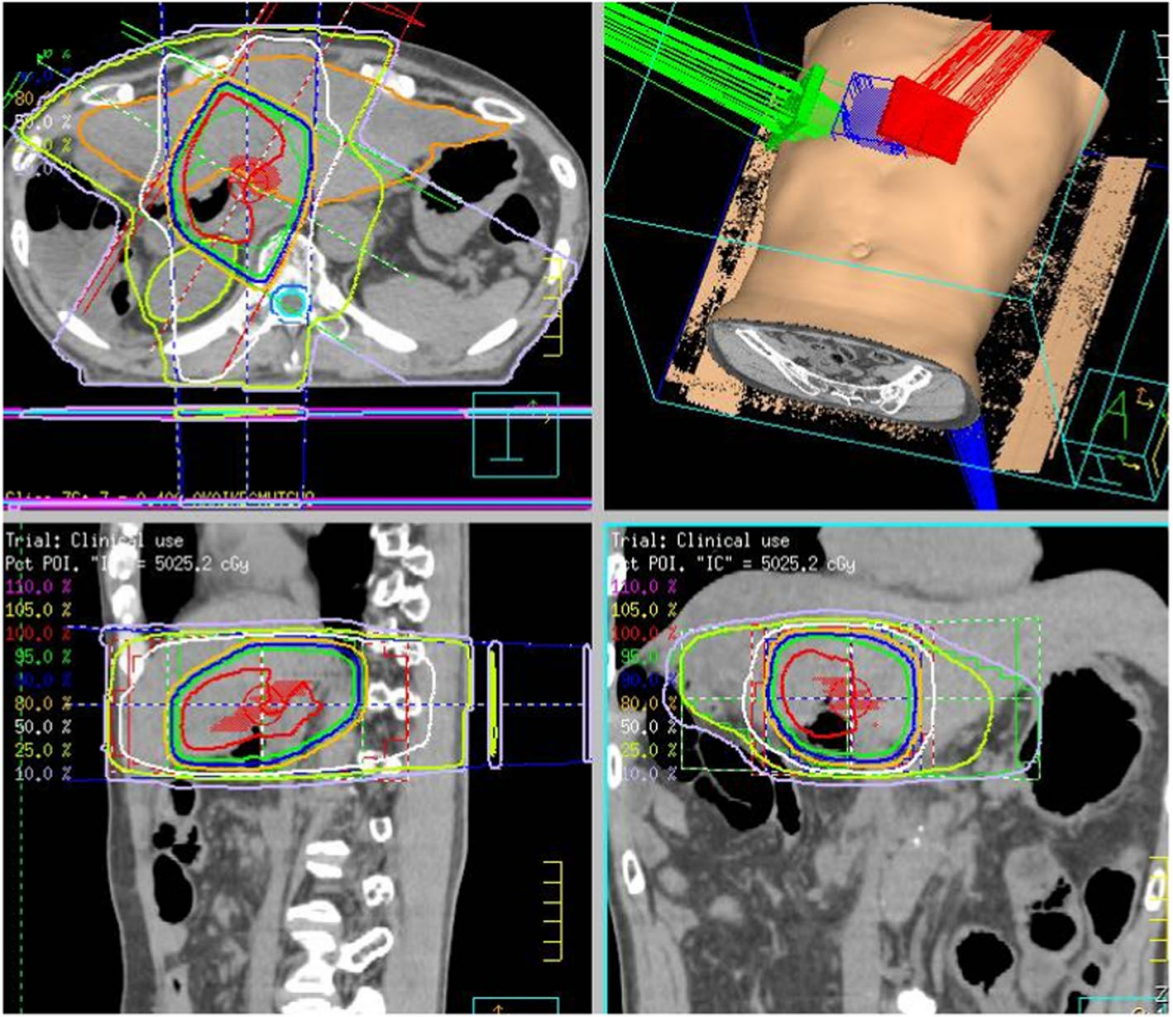
Radiotherapy planning summary. The root of the left hepatic vein was included in the irradiated field

A 62-year-old man with Bismuth type IIIa perihilar cholangiocarcinoma underwent right hemi-hepatectomy with caudate lobectomy and pancreatoduodenectomy (preoperative serum carcinoembryonic antigen: 2.5 ng/ml, carbohydrate antigen 19-9: 432 U/mL). After the management of postoperative complications of pancreatic fistula and refractory ascites, the patient was discharged on the 45th postoperative day. A histological examination of the resected specimen showed well-differentiated tubular adenocarcinoma (pathological T4N1M0, stage IIIB according to the Union for International Cancer Control classification of malignant tumors, 8th edition [6]). Although the resected specimen was grossly completely resected, microscopic findings revealed invasive carcinoma at the hepatic ductal margin. Therefore, adjuvant chemoradiotherapy with S-1 (S-1 80 mg/m^2^/day with an administered total of 28 days with 50.4 Gy/28 Fr) has performed from the third month after the operation (Fig. 1). Additional adjuvant chemotherapy with S-1 (S-1 80 mg/m^2^/day administered on days 1–28 every 6 weeks) was performed for 3 cycles, and the patient had no recurrence after surgery [2].

**Fig. 2:**
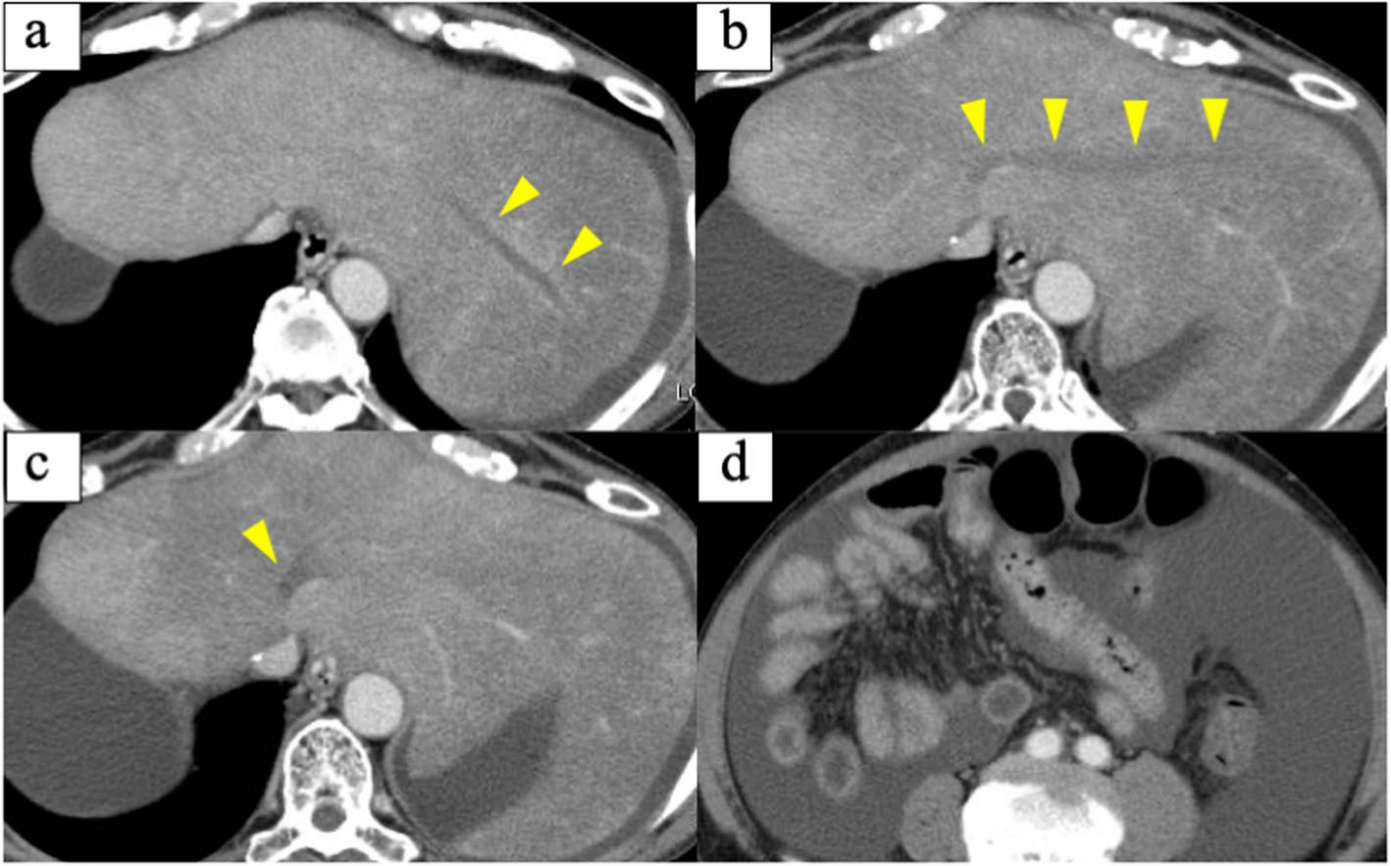
Multidetector-row computed tomography findings at the 32nd postoperative month. (before treatment for Budd-Chiari syndrome). **a**, **b**, **c** Yellow arrowheads show thrombotic occlusion of the LHV. **d** Massive ascites was confirmed

**Fig. 3:**
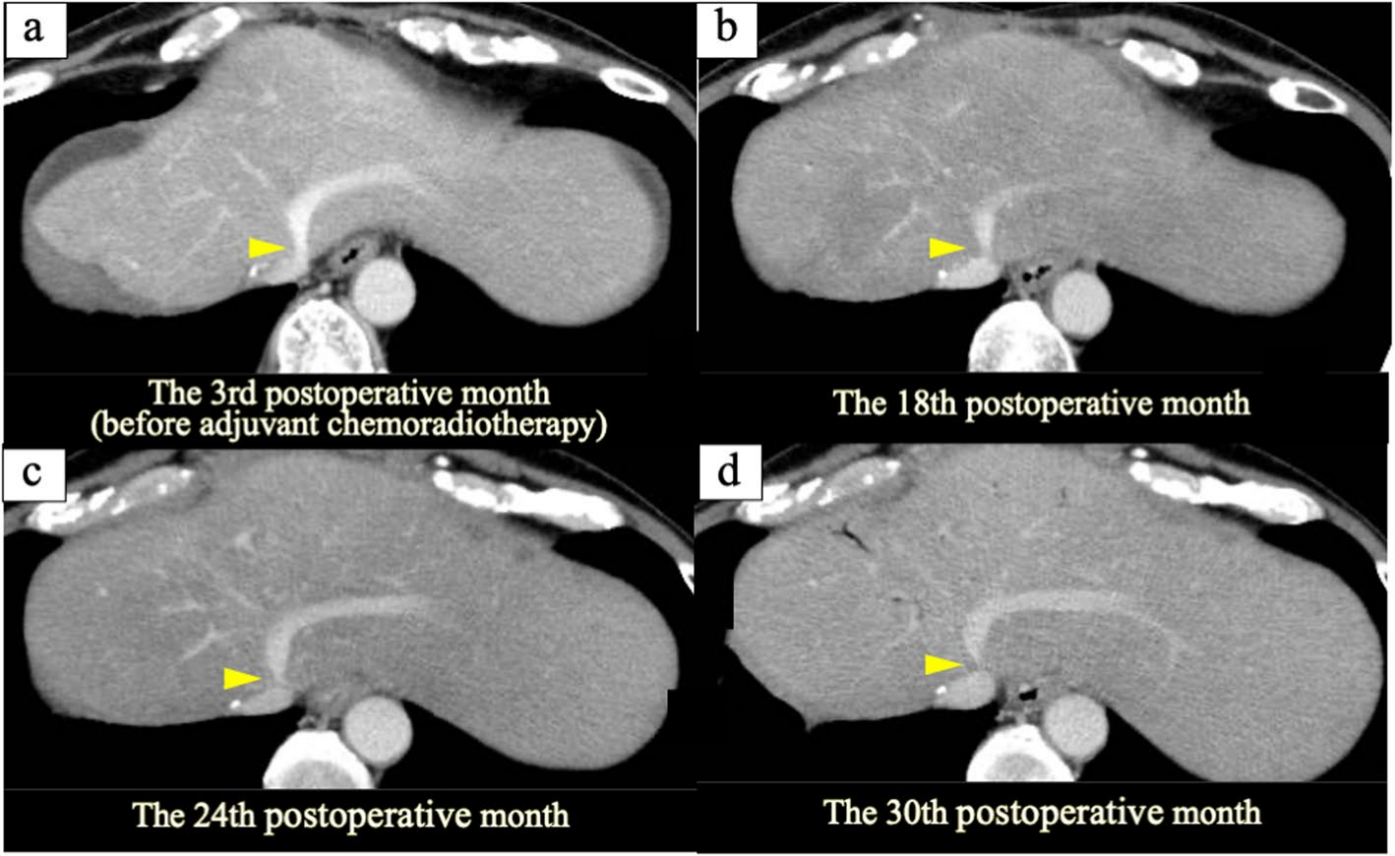
Multidetector-row computed tomography findings of the gradual narrowing process of the left hepatic vein. Yellow arrowheads show the root of the left hepatic vein. **a** The 3rd postoperative month. **b** The 18th postoperative month. **c** The 24th postoperative month. **d** The 30th postoperative month

The patient visited for acute onset abdominal distension and epigastric pain at the 32nd postoperative month. Elevated levels of serum aspartate aminotransferase and alanine aminotransferase were confirmed (Table 1), and multidetector-row computed tomography (MDCT) showed massive ascites and a contrast defect of the left hepatic vein (LHV) (Fig. 2). Cytology of ascites was negative for cancer and the serum-ascites albumin gradient was 1.3 g/dL (Table 1), suggesting the presence of portal hypertension due to hepatic congestion. Focusing on the LHV root, the part was gradually narrowed compared with the findings of MDCT before adjuvant chemoradiotherapy (Fig. 3). As the LHV root was included in the scope of radiotherapy (irradiated with about 46 Gy) (Fig. 1) and there were no surgical procedures that could have resulted in LHV root stenosis, it was determined that late side effects of radiotherapy had caused the LHV thrombotic occlusion. The diagnosis was therefore acute and secondary BCS caused by adjuvant radiotherapy.

**Table 1:** Blood test findings at the 32nd postoperative month (before anticoagulation therapy)

White Blood Cell	6750	/μL	**Total Protein**	**6.5**	**g/dL**	CEA	2.1	ng/mL
Red Blood Cell	489×10^4^	/μL	**Albumin**	**3.2**	**g/dL**	CA19-9	25.0	U/mL
Hemoglobin	12.9	g/dL	**AST**	**255**	**U/L**			
Hematocrit	39.4	%	**ALT**	**215**	**U/L**	**Complement value**	**49.4**	**CH50/mL**
Platelet	12.8×10^4^	/μL	**LDH**	**360**	**U/L**	**Antinuclear antibody**	**<40**	**TIMES**
			**ALP**	**177**	**U/L**	**Lupus anticoagulant**	**52.6**	**s**
**PT time**	**15.7**	**sec**	γ-GTP	69	U/L	**Protein-S % activity**	**41**	**%**
**PT % activity**	**54.0**	**%**	Cholinesterase	164	U/L	**Protein-C % activity**	**33**	**%**
PT-INR	1.42		CPK	136	U/L	Anti-Ro/SS-A antibody	Negative	
APTT	35.5	sec	BUN	13.3	mg/dL	Anti-La/SS-B antibody	Negative	
Fibrinogen	273.0	mg/dL	**Creatinine**	**1.08**	**mg/dL**	Anti-cardiolipin antibody	<8	U/mL
**FDP**	**7.8**	**μg/mL**	Total cholesterol	78	mg/dL	Anti-ds-DNA IgG antibody	<10	IU/mL
**D-dimer**	**2.4**	**μg/mL**	Total bilirubin	1.2	mg/dL			
**AT-III % activity**	**60**	**%**	**Direct bilirubin**	**0.7**	**mg/dL**	***Ascites analysis***		
			**C-reactive protein**	**4.67**	**mg/dL**	Appearance	Straw yellow	
			**Sodium**	**129**	**mEq/L**	Specific gravity	1.014	
			Potassium	4.9	mEq/L	White blood cell	120	/μL
			Chlorine	98	mEq/L	Albumin	0.9	g/dL

*PT* prothrombin, *CEA* carcinoembryonic antigen, *CA19-9* carbohydrate antigen 19-9, *FDP* fibrin degradation product, *AT* antithrombin, *LDH* lactate dehydrogenase, *ALP* alkaline phosphatase, *CPK* creatinine phosphokinase

Abnormal values are bolded

**Fig. 4:**
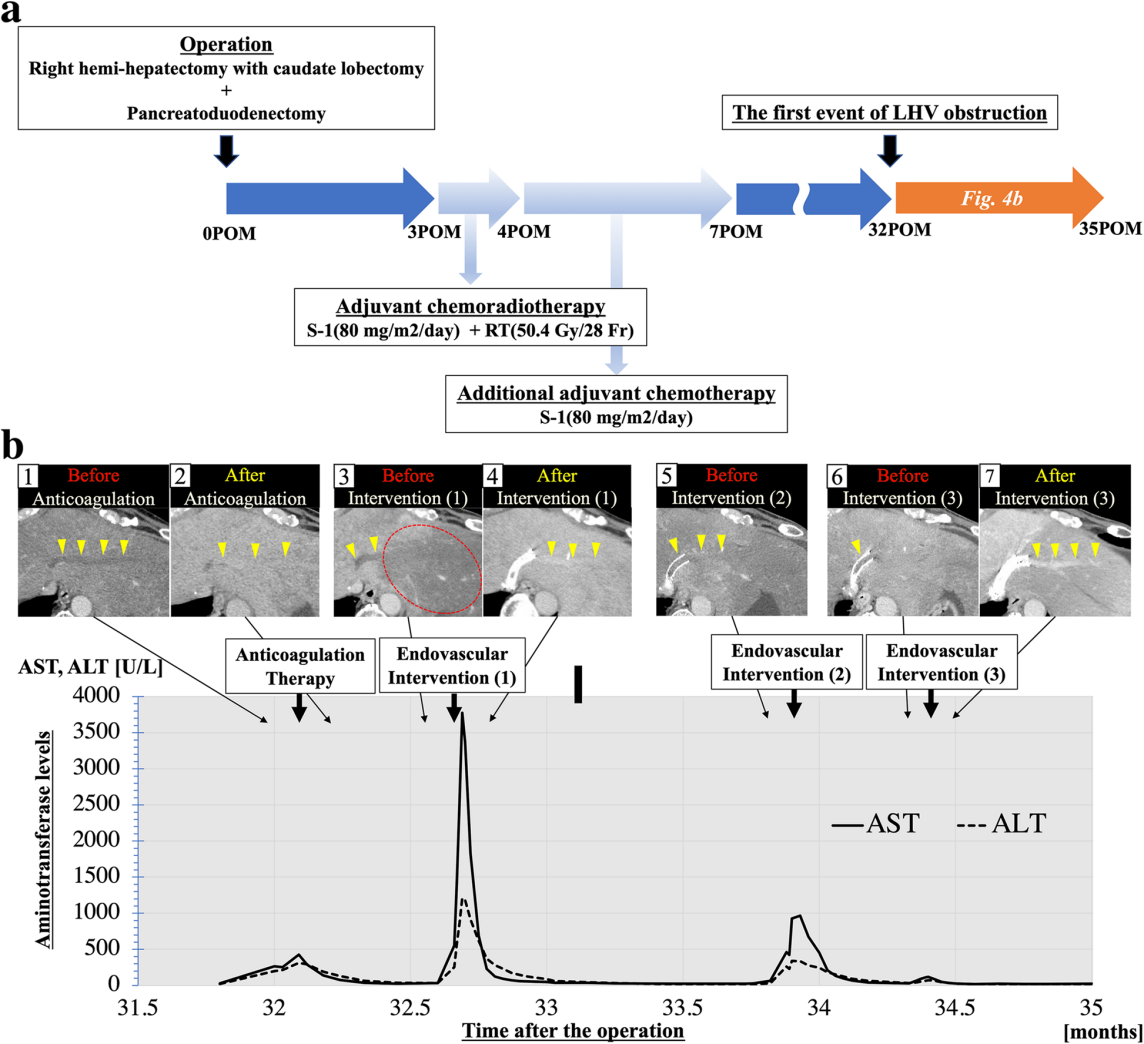
**a** An overview of the postoperative clinical course. **b** A time series showing multidetector-row computed tomography findings, the course of treatment, and the changes in serum AST and ALT levels. **b-1** Before the anticoagulation therapy. Yellow arrowheads show thrombotic occlusion of the LHV. **b-2** After the anticoagulation therapy. Yellow arrowheads show improvement in blood flow of the LHV. **b-3** Before the first endovascular intervention. Yellow arrowheads show thrombotic occlusion of the LHV. Hepatic congestion in the drainage area of the occluded LHV can be identified. **b-4** After the first endovascular intervention. Yellow arrowheads show improvement of blood flow of the LHV. **b-5** Before the second endovascular intervention. Contrast effects of the LHV were not observed. **b-6** Before the third endovascular intervention. Contrast effects of the LHV were not observed. **b-7** After the third endovascular intervention. Yellow arrowheads show improvement of blood flow of the LHV. POM, postoperative month; LHV, left hepatic vein; AST, aspartate aminotransferase; ALT, alanine aminotransferase

Figure 4a shows an overview of the postoperative clinical course, and Fig. 4b shows the treatment course for BCS. Anticoagulation therapy with heparin and administration of antithrombin III, which was insufficient, was started for reperfusion of the LHV. A slight reduction in the thrombus in the hepatic vein of the segment 2 (V2) was observed (Fig. 4b-2), and blood tests showed improvement of liver damage, with the ascites controlled with diuretics. However, after the 18th day of anticoagulation, the patient complained of sudden upper abdominal pain again, and the levels of serum AST and ALT were re-elevated (Fig. 4b). Furthermore, the prothrombin % activity fell to 8% due to progressive liver failure. MDCT showed an enlarged LHV thrombus and a congested and swollen lateral segment of the liver (Fig. 4b-3).

**Fig. 5:**
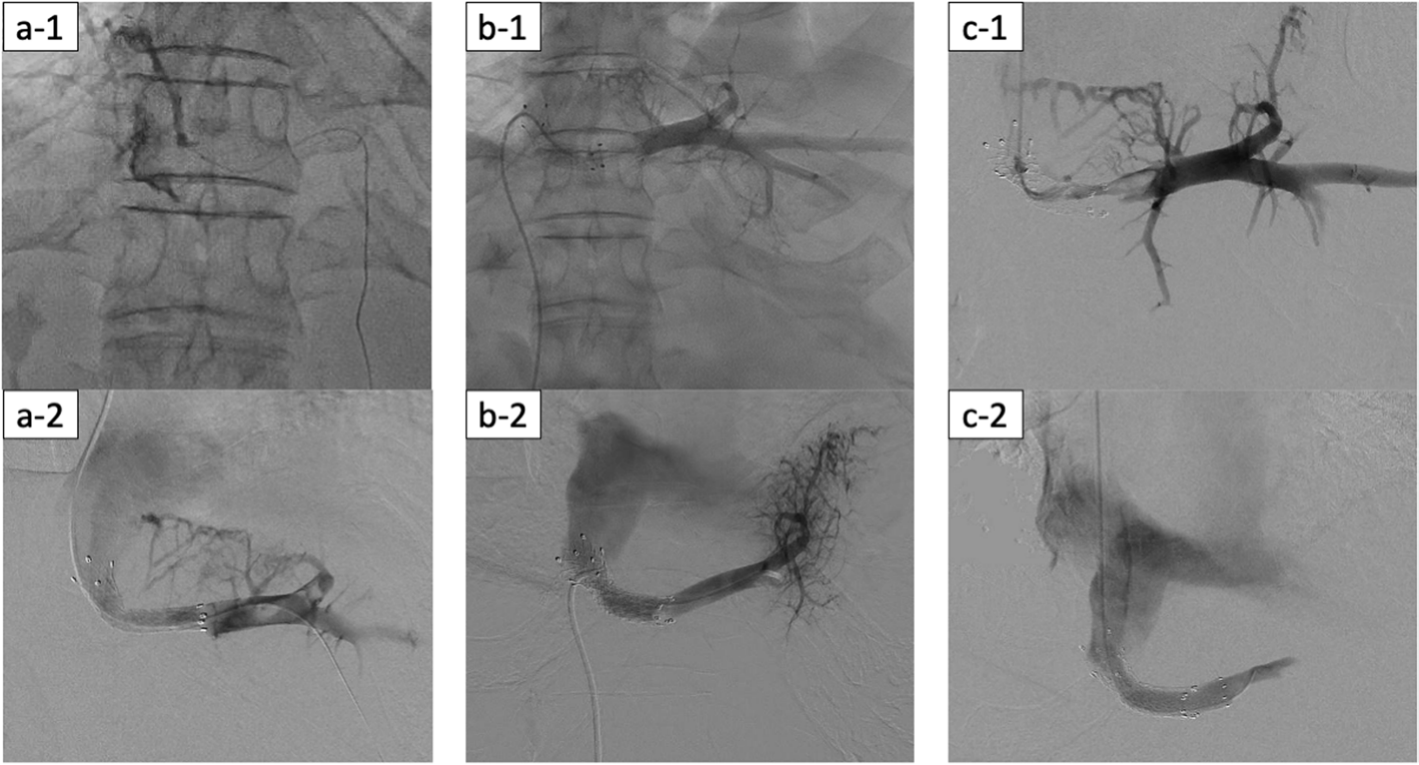
Findings of endovascular intervention. **a** The first time of intervention. Angiography of the V2 showing that the hepatic vein is completely occluded, with only collateral veins were contrasted (**a-1**). Improvement of blood flow of the LHV to IVC was confirmed after deployment of the LHV stent (**a-2**). **b** The second intervention. Angiography of the V2 showed no contrast effect from the V2 to IVC. (**b-1**) Improvement of the blood flow of the LHV to IVC was confirmed after deployment of the LHV stent (stent-in-stent) (**b-2**). **b** The third intervention. Angiography of the V2 showed marked stenosis in the existing stent (**c-1**). Improvement of the blood flow of the LHV to IVC was confirmed after deployment of the LHV covered stent (**c-2**). V2, hepatic vein of the segment 2; LHV, left hepatic vein; IVC, inferior vena cava

The patient was diagnosed with exacerbated acute BCS that might lead to fatal liver failure, so stenting was planned for the stenotic LHV root by endovascular intervention after correction of the severe hypocoagulable state by administration of 40 units of fresh-frozen plasma. Right internal jugular vein catheterization was performed under local anesthesia using the Seldinger technique. A 5-Fr diagnostic catheter (cobra type and hook type) was inserted through a 6-Fr sheath. Because the approach failed to cannulate the LHV, we added a transhepatic approach using a 21-gauge needle and established a pull-through route. Angiography of the V2 revealed the hepatic vein to be completely occluded (Fig. 5a-1). An 8 × 40-mm bare stent (E-LUMINEXX®; Bard Peripheral Vascular, New Providence, NJ, USA) was successfully deployed (Fig. 5a-2), and the tract in the hepatic parenchyma was embolized with a mixture of N-butyl-2-cyanoacrylate (NBCA) (Histoacryl; B. Braun, Melsungen, Germany) and iodized oil (lipiodol; Guerbet, Tokyo, Japan) (NBCA:lipiodol, 1:1).

The blood flow of the LHV showed improvement (Fig. 4b-4), and the abdominal pain symptoms rapidly improved after deployment of the stent, implying that hepatic congestion had improved. The subsequent clinical course was good with anticoagulant therapy (30 mg/day of edoxaban tosilate hydrate), and the patient was discharged 16 days after placement of the LHV stent.

One month after the placement of the LHV stent, the patient complained of sudden abdominal pain again. MDCT showed a loss of contrast effect in the LHV (Fig. 4b-5). It was considered that LHV thrombus occlusion had reoccurred, and a second angioplasty procedure was performed. An angiography of the V2 via the transjugular approach revealed no contrast effect from the V2 to the IVC (Fig. 5b-1). Although thrombolysis and balloon angioplasty were performed, the pressure gradient between the IVC and the LHV was still high. Another stent (E-LUMINEXX®; 10 × 40mm) was deployed in the existing stent, and the blood flow of the LHV showed improvement (Fig. 5b-2), while the pressure gradient was markedly decreased.

However, after the patient’s condition had recovered, a rapid increase in ascites was confirmed two weeks later. MDCT revealed a loss of contrast effect in the LHV (Fig. 4b-6), and we determined that LHV thrombus occlusion had occurred a third time. Angiography of the V2 via the transjugular route revealed marked stenosis in the existing stent (Fig. 5c-1). A 10 × 50-mm stent graft (Viabahn®; W. L. Gore, Flagstaff, AZ, USA) was successfully deployed in the second stent (Fig. 5c-2), and the blood flow of the LHV showed improvement (Fig. 4b -7). The LHV pressure was dropped to 20 mmHg from 60 mmHg. After stabilization of his condition, the patient was started on 100 mg/day of Aspirin and 60 mg/day of edoxaban tosylate hydrate.

As of 6 months since the last stenting, the patient is alive without restenosis of the LHV or recurrence of cholangiocarcinoma.

## Discussion

The present case describes acute and secondary BCS caused by adjuvant radiotherapy for perihilar cholangiocarcinoma following right hemi-hepatectomy and caudal lobectomy with pancreatoduodenectomy. Although a case of BCS caused by radiation-induced stenosis of the hepatic vein has not been reported, the present case showed that late side effects of radiation can cause hepatic vein stenosis and secondary BCS, and that stenting for radiation-induced stenosis of the hepatic vein might be an effective treatment.

Acute BCS is usually associated with severe symptoms, a poor prognosis, and high mortality and is rarer than chronic BCS, which has an insidious onset [5]. In addition, secondary BCS, which is caused by external factors such as invasion or extrinsic compression of the hepatic vein and/or the IVC, is rarer than primary BCS, which accounts for 60 to 70% of patients in Western countries [5]. In the present case, because there were no injuries due to a surgical procedure, including anastomosis [7], or postoperative infectious complications or recurrence that could have caused LHV root stenosis, it is natural to assume that radiotherapy caused the LHV stenosis. Therefore, although the present case involved a very rare situation, it was diagnosed as acute and secondary BCS induced by radiotherapy.

Radiation injury triggers inflammation and stimulates transdifferentiation of fibroblasts into myofibroblasts. Excessive proliferation of myofibroblasts produces excess collagen and other extracellular matrix components, and tissue compliance is reduced [8]. Radiation-induced fibrosis caused by these mechanisms can occur in any organ in the treatment field [9]. Particularly in the blood vessels, radiation-induced fibrosis can cause stenosis of the vessels [8, 10]. Although radiation-induced carotid stenosis after radiotherapy for head and neck cancer is well known [9], there are no reports concerning stenosis of the hepatic vein. In radiation-induced carotid stenosis, a previous report showed that the median interval between radiation therapy and intervention was 16 years [11]. Another previous report showed that intraoperative radiation therapy (20 Gy) during pancreatoduodenectomy for periampullary disease caused extrahepatic portal vein occlusion [10]. According to that report, 12 of 53 patients (22.6%) who underwent intraoperative radiation were diagnosed with extrahepatic portal vein occlusion, and the median period to the onset of extrahepatic portal vein occlusion was 358 days [10]. Although radiation-induced venous stenosis has rarely been described compared with radiation-induced arterial stenosis, it may be a serious complication [10, 12, 13]. In the present case, although stenosis of the LHV root was identified over time, symptoms appeared 28 months after the last adjuvant radiotherapy session. As the veins have a lower pressure system than the arteries, radiation-induced fibrosis of the veins and surrounding tissue may have affected to vessels more quickly than the arteries, such as the carotid artery.

Management of BCS involves four steps: anticoagulation therapy, angioplasty, and stenting for stenosis of the hepatic vein or the IVC, transjugular intrahepatic portosystemic shunts, and liver transplantation [14]. Previous reports have shown that endovascular interventions in BCS are associated with good long-term clinical outcomes and survival [14, 15]. Although the present case involved unique circumstances after right hemi-hepatectomy with caudate lobectomy and pancreatoduodenectomy, treatment was performed according to the usual BCS management approach. Stenting at the LHV root was very effective, as shown by the immediate improvement of abdominal pain after the deployment of the stent. However, restenosis occurred twice due to thrombus in the existing stent, and twice re-stenting was performed. Although the reason for the recurring stent thrombosis was unclear, an expanded polytetrafluoroethylene (ePTFE)-covered stent graft may have potential advantages in providing an inert and stable intravascular matrix for endothelization and a barrier for the development of intimal hyperplasia [16]. Therefore, an ePTFE-covered stent graft may be useful for managing treatment-resistant lesions after balloon angioplasty and bare stent placement. Indeed, the patient has shown no stent thrombus or restenosis of the LHV root as of 6 months since the last stenting. However, close follow-up will be needed, as tissue fibrosis after radiotherapy around the LHV root is expected to continue to progress.

## Conclusion

The present case is the first report of a case with BCS induced by radiotherapy. This case shows that late side effects of radiotherapy can cause hepatic vein stenosis and secondary BCS.

## Data Availability

The datasets used during the current study are available from the corresponding author upon reasonable request.

## Abbreviations

BCS: Budd–Chiari syndrome
IVC: Inferior vena cava
AST: Aspartate aminotransferase
ALT: Alanine aminotransferase
MDCT: Multidetector-row computed tomography
LHV: Left hepatic vein
V2: Hepatic vein of the segment 2
ePTPF: Expanded polytetrafluoroethylene
